# Effect of ruxolitinib on the oral mucosa in steroid-refractory graft-versus-host disease: a prospective observational exploratory study

**DOI:** 10.1007/s00784-025-06604-x

**Published:** 2025-11-06

**Authors:** Mar Bellido, Ana-Mae E. Jonk, Martina Kaurinovic, Anouschka Biswana, Carin L. E. Hazenberg, Goda Choi, Linde M. Morsink, Gerwin Huls, Arjan Vissink, Konstantina Delli

**Affiliations:** 1 Department of Hematology, University of Groningen, University Medical Center Groningen, Hanzeplein 1, Groningen, 9713 GZ the Netherlands; 2 Department of Oral and Maxillofacial Surgery, University of Groningen, University Medical Center Groningen, Hanzeplein 1, Groningen, 9713 GZ the Netherlands; 3 Center for Dentistry and Oral Hygiene, University of Groningen, University Medical Center Groningen, Groningen, the Netherlands

**Keywords:** Oral SR-cGVHD, Oral manifestations, Ruxolitinib, Lichen planus, Oral mucosa

## Abstract

**Objectives:**

To assess whether Steroid-Refractory chronic Graft-versus-Host Disease (SR-cGvHD) patients with oral involvement benefit from ruxolitinib regarding their clinical symptoms and oral lesions.

**Materials and methods:**

A prospective real-life observational longitudinal patient study was conducted in patients with SR-cGVHD and oral involvement who were treated with ruxolitinib. The effects of ruxolitinib on oral lesions and symptoms were assessed 12 weeks after the start of treatment with ruxolitinib based on the complete response, partial response, and overall response rate. Additionally, oral mucosal clinical characteristics and symptoms were assessed using validated outcome scales 4 and 12 weeks after treatment.

**Results:**

A total of 17 patients was included. Overall response rate after 12 weeks was 77%: 12% had a complete response and 65% had a partial response. A significant decrease was seen in presence of erythema (*p* = 0.002), lichenoid lesions (*p* = 0.001), median lesion site score (*p* = 0.001) and activity score (*p* = 0.001) after 12 weeks. Discomfort experienced by the patients in terms of mouth dryness, mouth pain, and mouth sensitivity was significantly ameliorated (*p* = 0.01).

**Conclusion:**

Treatment of oral SR-cGVHD with ruxolitinib ameliorates oral lesions and symptoms experienced by patients.

**Clinical relevance:**

: Ruxolitinib effectively treats symptoms of oral cGHVD as shown by real world evidence.

**Supplementary Information:**

The online version contains supplementary material available at 10.1007/s00784-025-06604-x.

## Introduction

Hematopoietic stem cell transplantation (HSCT) is a curative treatment for various hematological diseases [1], with over 43,000 patients undergoing the procedure annually in Europe [2]. However, 30–40% of these patients develop Graft-versus-Host Disease (GVHD) [3], in which the transplanted donor cells trigger an immune response against the recipient’s normal cells [4]. GVHD can manifest as acute (aGVHD), typically affecting the gut, liver and skin or chronic (cGVHD) disease, which may involve various organs, mainly the skin, eyes, lungs, joints and oral mucosa [5].

The pathogenesis of cGVHD involves immune dysregulation leading to inflammation and tissue damage, followed by abnormal tissue repair and fibrosis [6, 7]. Various factors, such as donor-recipient sex mismatch, older donor for recipient, human leukocyte antigen (HLA) mismatch and history of acute GVHD, predispose individuals to cGVHD [5]. A high percentage of patients with cGVHD develop oral involvement (45–83%), presenting with erythema, ulcerations, lichenoid lesions and xerostomia. These manifestations can severely impact - patient’s quality of life by hindering daily activities, and are associated with an increased risk of oral infections, - mucoceles, and oral cancers [5].

For patients requiring systemic immunosuppression, first-line treatment typically consists of systemic corticosteroids, often in combination with a calcineurin inhibitor [8, 9]. Mild, limited cGVHD (e.g., grade 1 skin or grade 1 oral) is frequently managed with topical therapies alone [8, 9]. However, - approximately 53% of patients do not respond to this therapy, resulting in steroid-refractory (SR) cGVHD [10]. Second-line treatment options include ruxolitinib, an oral Janus Kinase (JAK) 1/2 inhibitor which has shown promising results in treating organs affected by SR-cGVHD [11–20]. Although ruxolitinib has been approved by both the FDA and EMA as a treatment for SR-cGVHD and steroid-dependent cGVHD, its specific effects on the oral mucosa have not been extensively studied. Previous retrospective studies have shown encouraging outcomes, with a high response rate in patients with oral lesions [15, 21].

In the prospective, phase 3 randomized trial REACH 3 [22], ruxolitinib demonstrated a significant better symptom response in all organs compared to the control group. However, to fully understand the response of the oral mucosa to ruxolitinib in SR-cGVHD patients, it is essential to assess the experienced symptoms, as well as the clinical activity, severity, and extent of oral lesions.

Therefore, the primary aim of the current study was to prospectively explore the effect of ruxolitinib on the oral mucosa in a well-defined, real-world cohort of - SR-cGVHD patients with oral involvement, based on the 2014 NIH consensus criteria for complete response (CR), partial response (PR) and overall response rate (ORR) [23] after 12 weeks of ruxolitinib treatment. Additionally, various validated scales were employed to assess additional parameters related to the oral mucosa and patient-reported outcomes after 4 and 12 weeks of therapy.

## Materials & methods

### Study design

This prospective longitudinal observational study focused on patients with SR-cGVHD who received ruxolitinib as part of their routine treatment at the Department of Hematology, University Medical Center Groningen (UMCG). The study builds on the findings of Kaurinovic et al., who previously reported outcomes in a retrospective cohort [21]. The current study aimed to assess changes in the oral mucosa over time, comparing baseline observations to those at 4 and 12 weeks using multiple validated scoring scales. Ethical approval was obtained from the Medical Ethics Review Committee of the UMCG (M19.232532).

## Study population

The research population consisted of 17 consecutive patients diagnosed with SR-cGVHD and oral manifestations at the UMCG between October 2019 and June 2022. All these patients received ruxolitinib as a second-line standard therapy for SR-cGVHD. Patients with extensive cGVHD affecting organs other than the oral mucosa were treated with systemic prednisolone and a calcineurin inhibitor as first- line therapy, and these drugs were continued when ruxolitinib was introduced as second- line treatment. Patients with localized cGVHD organ involvement (1 or 2 organs) were treated with local therapies and, if refractory, local treatment was discontinued and ruxolinib was administrated as a second-line therapy.

Inclusion criteria encompassed patients with isolated oral SR-cGVHD as well as those with oral SR-cGVHD in combination with-cGVHD in other organs. All patients were aged 18 or older. Patients were excluded if they received ruxolitinib for indications other than SR-cGVHD or were unwilling or unable to provide informed consent. Eligible patients with extensive cGVHD organ involvement outside the oral cavity had previously received first-line treatment with oral prednisolone and a calcineurin inhibitor (cyclosporin or tacrolimus), along with topical dexamethasone, triamcinolone, or clobetasol for oral manifestations, as - prescribed following their visit to the Oral Medicine outpatient clinic of the UMCG.

Ruxolitinib was introduced due to a lack of response to first-line treatment, and the dosage was 5 mg or 10 mg orally administered twice daily, depending on the severity of cGVHD. A power analysis based on data from the retrospective study of Kaurinovic et al. (2022) indicated an sample size of 17 for the current study (effect size = − 0.66; α error probability = 0.05; Power (1-β error probability) = 0,8) (G*Power Heinrich-Heine Universität, Düsseldorf, Germany).

## Variables & methods

Oral mucosa involvement was assessed using the following scales to analyze the effect of ruxolitinib:

- **NIH Modified Oral Mucosal Rating Scale**, in which erythema and lichenoid lesions are each scored 0–3, and ulcerations are scored 0–6 (3 = moderate; 6 = severe). The composite score (sum of the three components) ranges from 0 to 12 [23].;
- **Escudier Scale**, which assesses the location of lesions in the oral cavity and the severity of each lesion (both on a scale from 0 to 24), as well as the overall lesion activity on a scale from 0 to72 [24];
- John Hopkins Mouth Oral Score, which measures perceived oral pain on a scale from 0 to3 [25];
- **NIH Esophagus Score**, which evaluates symptoms of dysphagia and odynophagia on a scale from 0 to 3 [23];
- **NIH Chronic GvHD Global Severity Score**, which assesses the overall severity of cGVHD in the individual patient- on a scale from 0 to3 [26];
- **NIH Oral Symptom Score**, which evaluated patient-reported mouth dryness, mouth pain, and mouth sensitivity on a scale from 0 to10 [27].

The same procedure was conducted at baseline, 4 weeks, and 12 weeks following the initiation of treatment. Scoring was performed by a trained observer (A.M.J.), who had received prior instruction in assessing oral mucosa lesions. Additionally, the observer captured photographs of the oral mucosa for each patient during every visit. In cases of uncertainty regarding the scoring, the observer consulted senior clinicians (A.V. and K.D) for guidance.

Scores of 0 indicated the absence of symptoms, while higher scores reflected increasing symptom severity. The 2014 NIH consensus criteria were followed to define complete response (CR) and partial response (PR) [23]. CR was defined as the complete resolution of symptoms, whereas PR was defined as an improvement of at least 1 point on a 4-point scale or 2 points on a 12-point scale. Together, CR and PR constituted the overall response rate (ORR). To ensure anonymity, patients were assigned Unique Patient Numbers, and data were collected and stored in Microsoft Excel v.16.36 (2020) (Microsoft Corporation, Redmond, New York, USA) within the secure digital environment of the UMCG.

Furthermore, data were gathered on patients’ gender and age, the dosage of ruxolitinib administered, any additional immunosuppressive therapy required or initiated during the research period, and the use of topical treatments throughout the study.

Since this was a real-life cohort, patients continued to be monitored in routine clinical practice beyond the initial 12-week period. No additional visits, assessments, or interventions were scheduled as part of the study. Data obtained after 12 weeks were derived solely from standard clinical follow-up, without any deviation from routine care. These data were included post hoc to provide a broader perspective of patient outcomes.

### Statistical analysis

After verifying the absence of normality through visual examination of histograms for the interval scales, the Friedman test was conducted for all scales, followed by the sign test as a post-hoc analysis. A p-value ≤ 0.05 was considered statistically significant for each scale. Data analysis was performed using IBM SPSS Statistics 28 (IBM, Armonk, New York, United States).

Additionally, a statistical model was built using Generalized Estimating Equations (GEE) to analyze the efficacy of ruxolitinib after 4 and 12 weeks. The confounding variables considered included gender, age, ruxolitinib dose, immunosuppressive therapy, topical therapy and baseline score. Different correlation structures (exchangeable, M-dependent, unstructured) were tested, and the model with the lowest information criterion was selected, which was the exchangeable correlation structure for all variables.

## Results

### Patient characteristics

Between October 2019 and June 2022, 23 consecutive patients suspected of having SR-cGVHD were screened, of which 17 started treatment with ruxolitinib. Six patients did not initiate ruxolitinib treatment -, either because they were diagnosed with SR-cGvHD in organs other than the oral mucosa (*n* = 5); or different treatment was given (*n* = 1). All 17 patients who started ruxolitinib were suitable for analysis. Table 1 shows the demographic characteristics of these patients in more detail. Supplementary Table 1 provides additional baseline clinical characteristics including previous history of aGvHD, organs involved and previous immunosuppressive therapy. No new topical or local agents for oral cGvHD were initiated during the observation period.

**Table 1:** Characteristics of patients included in this research

Characteristic	*n*	%
Patients	17	100
Gender:		
Female	6	35.3
Male	11	64.7
Age (years):		
Median	64.7	
Range	26.3–76.8	
Ruxolitinib (dose):		
5 mg twice daily	11	64.7
10 mg twice daily	6	35.3
Immunosuppressants usage during research period:		
Yes	12	70.6
No	5	29.4
Start new immunosuppressants during research period:		
Yes	4	23.4
No	13	76.5
Local treatment during the research period:		
Yes	10	58.8
No	7	41.2
cGvHD Global Severity Score (mean, IQR)	2	2–2

## Oral mucosa changes after treatment with ruxolitinib

After 12 weeks of treatment, in total 5 (30%) patients achieved a CR and 12 (70%) patients achieved a PR, resulting in a 100% ORR. Of these patients, 13 were treated exclusively with ruxolitinib for SR-GvHD, while 4 patients required additional systemic immunosuppressive treatment, due to cGVHD involvement in organs other than to the oral mucosa.

Among the patients treated exclusively with ruxolitinib, 2 (11.8%) achieved a CR, and 11 (64.7%) achieved a PR resulting in an ORR of 76.5% at 12 weeks. The 4 patients treated with ruxolitinib in combination with additional systemic immunosuppressants were excluded from the ORR analysis. However they were still included in the analysis and interpretation of oral symptoms using various validated scales, as the additional immunosuppressants were added for reasons unrelated to the response of the oral mucosa.

## Changes of oral symptoms after treatment with ruxolitinib

**Fig. 1:**
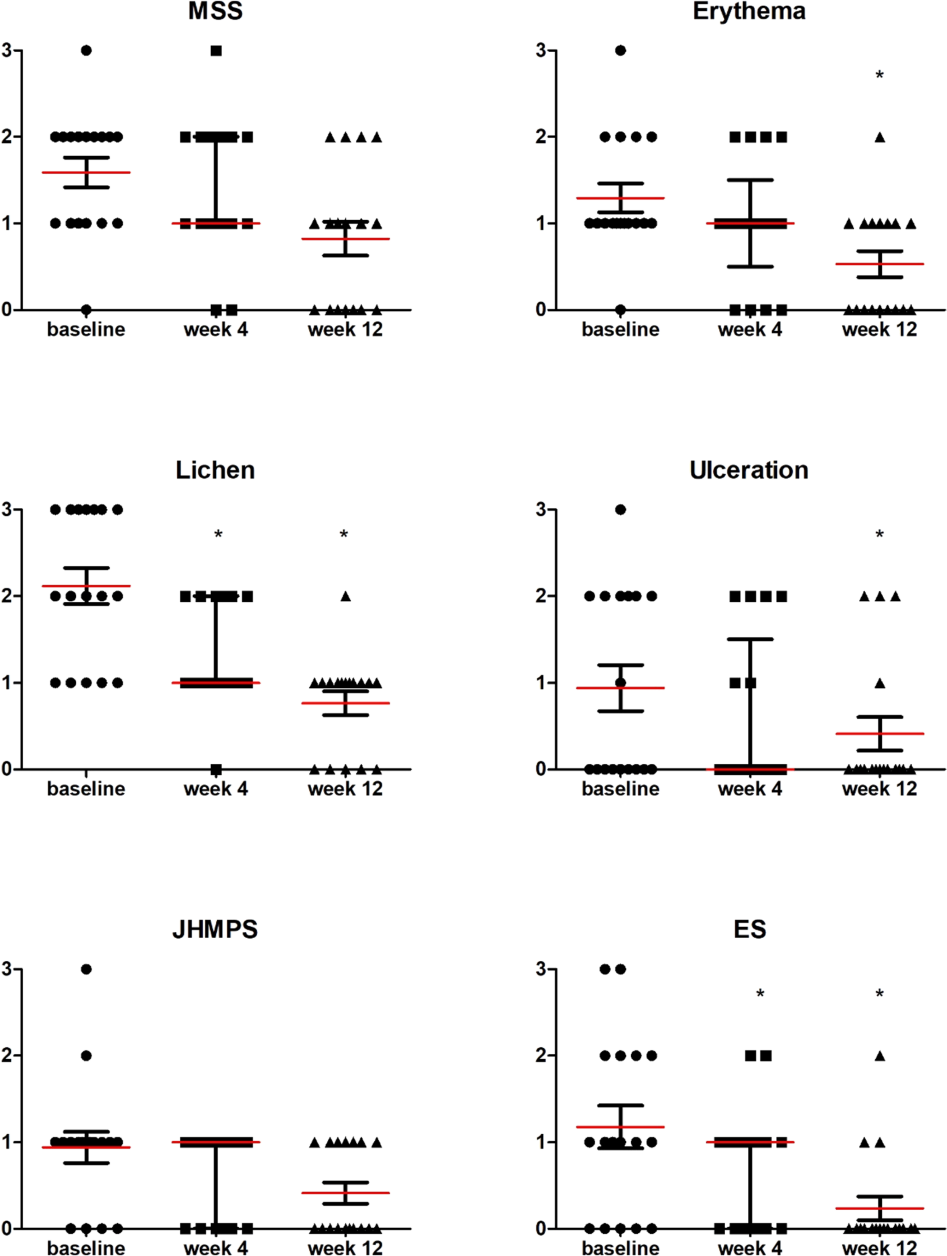
Median score per scale at baseline, 4 weeks, and 12 weeks (*N* = 17) after starting ruxolitinib * Significant difference from baseline score (*p* < 0.05). MSS: Mouth Staging Score; JHMPS: John Hopkins Mouth Pain Score; ES: Esophagus Score. Red horizontal lines indicate median values

**Fig. 2:**
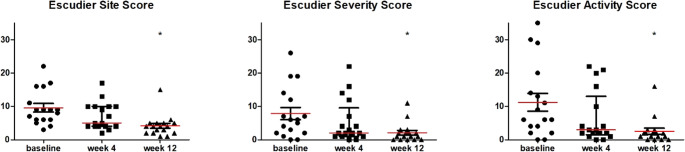
Median per total score of the Escudier Scale at baseline, 4 weeks, and 12 weeks (*N* = 17) after starting ruxolitinib. Site and severity scores range from 0–24 and activity scores from 0–72, where 0 = no symptoms. * Significant difference from baseline score (*p* < 0.05). Red horizontal lines indicate median values

**Fig. 3:**
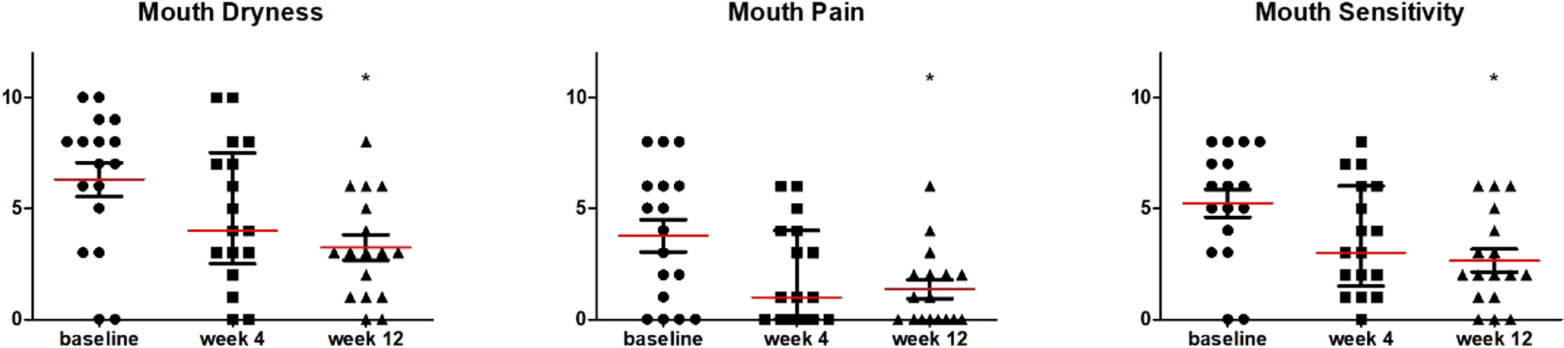
Median scores per symptom score according to the NIH Oral Symptom Scores at baseline, 4 weeks, and 12 weeks after the start of ruxolitinib. Scores range from 0 (no symptoms) to 10 (as bad as one can imagine). Red horizontal lines indicate median values. * indicates statistical significant difference compared to baseline

All scales showed a reduction in symptoms after 12 weeks of ruxolitinib treatment (Figs. 1, 2 and 3). Figure 1 displays the median scores per scale at various time points. Early improvement in oral signs after 4 weeks of treatment was seen for lichenoid lesions and the esophagus score. After 12 weeks of treatment, further significant improvements were noted in erythema, lichenoid lesions, ulcers and the esophagus score (Table 2). A significant decrease in the median score for lesion sites on the oral mucosa was observed at 12 weeks compared to baseline (Fig. 2). Furthermore, the median lesion severity score and lesion activity score decreased over the 12-week period.

**Table 2:** Scales with a significant outcome

Scoring scale	Focus	Time	Median	Interquartile range	*p*
NIH Oral Mucosa Rating Scale	Erythema	Baseline 4 weeks 12 weeks	1 1 0	2–1 1.5–0.5 1–0	*> 0.05* *0.002*
	Lichenoid lesions	Baseline 4 weeks 12 weeks	2 1 1	3–1 2–1 1–0	*0.001* *0.001*
	Ulcers	Baseline 4 weeks 12 weeks	0 0 0	2 − 0 1.5-0.5 0.5-0.5	*> 0.05* *> 0.05*
NIH Esophagus Score	Dysphagia & odynophagia	Baseline 4 weeks 12 weeks	1 1 0	2–0 1–0 0–0	*0.02* *0.001*
Escudier Scale	Site	Baseline 4 weeks 12 weeks	6 5 4	13.5–6 10–4 5–2	*> 0.05* *0.001*
	Severity	Baseline 4 weeks 12 weeks	6 2 1	13.2–2 9.5–1 2.5–0	*> 0.05* *0.001*
	Activity	Baseline 4 weeks 12 weeks	6 3 2	17–3 13-1.5.5 2.5–0.5	*> 0.05* *0.001*
NIH Oral Symptom Score	Mouth dryness	Baseline 4 weeks 12 weeks	7 4 3	8.5–4 7.5–2.5 5.5–1	*> 0.05* *0.001*
	Mouth pain	Baseline 4 weeks 12 weeks	4 1 1	6–0.5 4-0 4.5–1	*> 0.05* *0.01*
	Mouth sensitivity	Baseline 4 weeks 12 weeks	6 3 2	7.5–3.5 6–1.5 4.5–1	*0.01* *0.001*
NIH Chronic GvHD Global Severity Score		Baseline 4 weeks 12 weeks	2 2 2	2–2 2 − 1 2.5-1.5	*> 0.05* *> 0.05*

The assessment of discomfort using the NIH Oral Symptom Score revealed significant improvements in mouth dryness, mouth pain and mouth sensitivity (Table 2; Fig. 3), with all parameters showing significant improvement from baseline to 12 weeks.

**Fig. 4:**
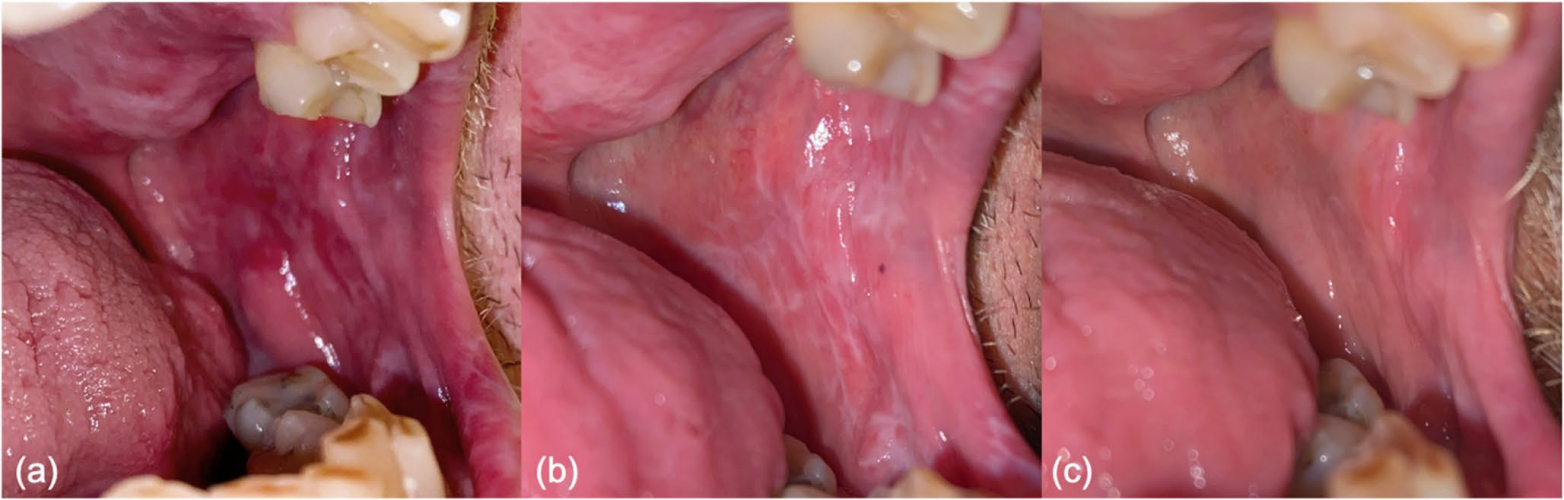
Clinical presentation of the left buccal mucosa. (**a**) At baseline. (**b**) At 4 weeks. (**c**) At 12 weeks

A representative example of a single patient showing clinical findings in the oral mucosa at baseline, 4 weeks, and 12 weeks is shown in Fig. 4. Gender, age, ruxolitinib dose, concomitant systemic immunosuppressive therapy, use of topical therapy, and baseline oral cGvHD scores did not show a statistically significant association with treatment response at either 4 or 12 weeks following initiation of ruxolitinib (data not shown).

Of the 17 patients included within the initially predefined inclusion period, an additional fourth assessment of the oral mucosa status was performed in 11 cases at the final follow-up (ranging from − 8 to 36 months after the 12-week assessment).

At 12 weeks, all 17 evaluable patients demonstrated either partial (*n* = 12) or complete responses (*n* = 5) of oral cGvHD symptoms. With longer follow-up, 4 of the patients who initially achieved a partial response subsequently converted to complete remission, while the remainder maintained stable partial responses. Durable complete remissions of up to 36 months were observed. At the last follow-up (July 2022), most patients with sustained responses were no longer receiving ruxolitinib, indicating that clinical benefit could be maintained after treatment discontinuation. Overall, these data suggest that ruxolitinib induces durable responses in oral cGvHD, with the potential for ongoing remission after cessation of therapy.

Detailed results from this fourth assessment are shown in Table 3.

**Table 3:** Long-term overview of CR and PR responses and current use of ruxolitinib. * Patient deceased, ** Long-term effects not measured yet, UPN: unique patient number

UPN	Global Severity Score at 12 weeks	State oral symptoms at 12-weeks	Current, long term state oral symptoms	Time passed after 12-week check-point (months)	Currently (July 2022) still using ruxolitinib
1	3	CR	PR	32	Yes
2	2	PR	PR	16	No
3	2	PR	PR	15	Yes
4	1	PR	PR	12	Yes
5	1	PR	CR	11	No
6	1	PR	CR	10	No
7	1	PR	PR	8	Yes
8	2	PR		*	
9	2	PR		*	
10	2	PR	PR	33	No
11	3	CR		*	
12	2	CR	CR	35	No
13	3	PR	CR	36	No
14	2	PR	CR	33	No
15	1	PR		**	
16	1	CR		**	
17	3	CR		**	

In 6 patients this assessment could not be performed because the patient was either deceased (*n* = 3) or because insufficient time had passed between the additional check and the last official measurement (*n* = 3). During this fourth assessment, 4 patients who had achieved a PR after 12 weeks of ruxolitinib treatment went to achieve a CR following extended follow-up.

## Discussion

This study, which is builds upon the findings of the retrospective study of Kaurinovic and colleagues [21], prospectively evaluates the effect of ruxolitinib on alleviating oral clinical manifestations and symptoms experienced by patients with oral SR-cGVHD. It was shown that both clinical findings and self-reported symptoms associated with SR-cGVHD - significantly decreased 12 weeks after initiating ruxolitinib.

Only a few studies have specifically examined the effect of ruxolitinib on the oral mucosa in patients with SR-cGVHD. A meta-analysis by Fan et al. reviewed 8 retrospective studies and reported an ORR of 76.5% and a CR of 34% [28]. In comparison, our study showed an ORR of 100% with 30% of patients achieving a CR. Fan et al. also reported that ruxolitinib showed superior efficacy over other agents for both SR-aGVHD and SR-cGVHD, consistent with findings in the literature [16, 17]. The ORR was highest in SR-cGVHD affecting the oral mucosa, followed by other organ systems as skin, gut, joints and fascia, liver, eyes, esophagus, and lungs [28].

When comparing ruxolitinib with other therapies targeting oral mucosa involvement, it appears to be more effective than belumosudil, a small molecule inhibitor of Rho-associated coiled-coil kinase 2 (ROCK2). Patients treated with belumosudil reported an ORR of 55% [29], significantly lower than the 100% ORR observed in our study. Ibrutinib, a Bruton tyrosine kinase (BTK) inhibitor, demonstrated an ORR of 88% for oral mucosa involvement, which is also lower that the ORR observed with ruxolitinib in our study [30]. For imatinib, a BCR-ABL1 tyrosine-kinase inhibitor, Parra Salinas et al. reported a 36% improvement rate; however, the methodology used for this assessment was not clearly specified [31].

It is important to note that not all patients included in the above-mentioned referenced novel agent trials had oral cGvHD. Therefore, conclusions regarding their efficacy specifically on oral manifestations should be drawn with caution [32].

When assessing the possible influence of confounders, our study indicates that men experienced greater symptom improvement than women (data not shown). Although this difference was not statistically significant, a similar trend was also observed in the retrospective study by Kaurinovic et al. [21]. These findings suggest that women may experience more intense symptoms than men, despite similar pharmacokinetics between the genders. Previous studies have shown that women tend to report more severe xerostomia [12]. In our study, we did not assess hyposalivation, which may be an important factor contributing to differences in symptom severity between men and women. Additionally, while no comparable studies have specifically address gender differences in ruxolitinib treatment for SR-cGVHD, this remains an area for future exploration.

Our study also examined the effects of different ruxolitinib dosages. We found that patients taking 10 mg oral ruxolitinib 2 times a day did not achieve superior outcomes compared to those taking 5 mg twice daily. The pivotal trial by Zeiser et al. [22] established the efficacy of 10 mg twice daily was effective in treating SR-cGVHD, providing the FDA-approved starting dose for patients aged 12 years and older with SR-cGVHD [33]. However, it did not evaluate lower doses. Our finding should therefore be interpreted as exploratory. Nonetheless, given frequent dose reductions in clinical practice, these data suggest that lower doses may be sufficient for oral cGVHD symptoms in some patients and merit prospective confirmation.

To our knowledge, no previous real-world prospective study has comprehensively documented oral clinical manifestations, symptoms, and patient reported experiences following ruxolitinib treatment for SR-cGVHD to the extent achieved in this study. Moreover, the majority of our patients were followed beyond 12 weeks after initiating ruxolitinib. During the fourth additional assessment, four more additionally patients achieved a CR.

While our study provides valuable insights, several potential limitations must be considered. Variability in the type and dosage of immunosuppressants, the dosage of ruxolitinib, and the use of various local mouthwashes complicates attributing observed improvements solely to ruxolitinib. In an effort to address this issue, potential confounders were analyzed using a Generalized Estimating Equations (GEE) Model. In addition, we did not administer the Lee cGVHD Symptom Scale (a validated multisystem patient-reported outcome). Because this study focused specifically on oral cGVHD, we prioritized clinician-reported oral activity (NIH Modified Oral Mucosal Rating Scale) and objective oral endpoints. Another limitation is the relatively small sample size. While the required sample size was reached based on our power calculation, a larger cohort would have been desirable to improve the generalizability of our findings and allow for more extensive subgroup analysis. Unfortunately, recruitment was paused due to the COVID-19 pandemic, which limited further enrollment.

### Implications for future research

Several factors are known to increase the risk of developing cGVHD [32]. Future research should explore whether ruxolitinib can also prevent GVHD, in addition to treating it, particularly its potential prophylactic effects on oral SR-cGVHD. While early use of ruxolitinib has showed promising results in preventing aGVHD [34, 35], its role in preventing oral cGVHD remains unexplored. In the study by Zhao et al., (2020) two patients developed moderate or severe cGVHD after tapering or discontinuing ruxolitinib, resulting in a 1-year cumulative incidence of moderate or severe cGVHD of 21.4%. Three additional phase II studies are currently underway to investigate JAK inhibitors (including ruxolitinib) for GVHD prevention [36]. To date, no prophylactic studies of ruxolitinib have specifically focused on oral (SR-)cGVHD. Additionally, combining ruxolitinib with other immunosuppressants may enhance its effectiveness as a first-line treatment. Future studies could also incorporate saliva testing to objectively assess hyposalivation, potentially explaining gender differences in symptom severity.

In conclusion, ruxolitinib reduces the symptoms of oral mucosa involvement and alleviates the discomfort from dry mouth, mouth pain and mouth sensitivity in patients with SR-cGVHD. Ruxolitinib is a viable alternative therapeutical option and shows favorable effects in the treatment of oral lesions associated with SR-cGVHD.

## Supplementary Information

Below is the link to the electronic supplementary material.

ESM1 (DOCX 17.9 KB)

## Data Availability

Upon request.
