# Supplementary material for: Effect of ruxolitinib on the oral mucosa in steroid-refractory graft-versus-host disease: a prospective observational exploratory study
Source: Clin Oral Investig. 2025 Nov 6;29(11):552. doi: 10.1007/s00784-025-06604-x (PMC12589367; doi:10.1007/s00784-025-06604-x)
Supplement: Supplementary file 1 — (DOCX 17.9 KB) [file 784_2025_6604_MOESM1_ESM.docx]

# Supplementary Table 1: Additional baseline clinical characteristics of the patients

SIB: Sibling Donor; MUD: Matched Unrelated donor

*Abbreviations: SIB – Sibling Donor; MUD – Matched Unrelated Donor; aGVHD – Acute Graft-versus-Host Disease.*

| **UPN** | **Type of Donor** | **HLA match** | **Prior aGVHD**  **(Glucksberg grading)** | **cGVHD: organs involved (additionally to**  **Oral Cavity)** | **Prior History of Immunosuppressive Therapy** | **Ongoing immunosuppressive therapy at baseline** |
| --- | --- | --- | --- | --- | --- | --- |
| 1 | SIB | 10/10 | aGVHD grade II  skin, liver | Skin | Oral prednisolone, tacrolimus | - |
| 2 | MUD | 10/10 | aGVHD grade I  skin | Skin | Clobetasol cream,  Triamcinolone acetonide  cream, Dexamethason mouthwash | - |
| 3 | SIB | 10/10 | - | Skin, eyes | Tacrolimus mouthwash,  Cyclosporine eye drops | - |
| 4 | SIB | 10/10 | - | Liver | Tacrolimus mouthwash,  Oral cyclosporine | Oral cyclosporine |
| 5 | MUD | 10/10 | - | Eyes | Dexamethason mouthwash,  sodium hyaluronate eye drops | - |
| 6 | SIB | 10/10 | - | Genital | Clobetasol mouthwash, cream | - |
| 7 | SIB | 12/12 | aGVHD grade I  skin | Eyes | Dexamethason mouthwash,  sodium hyaluronate eye drops | - |
| 8 | MUD | 10/10 | - | Skin | Oral prednisolone tacrolimus,  Clobetasol mouthwash | - |
| 9 | MUD | 8/10 | aGVHD grade II  Skin | Skin | Triamcinolone acetonide  cream, oral prednisolone | - |
| 10 | MUD | 10/10 | aGVHD grade I  Skin | Skin, genital | Triamcinolone, mometasone  ointment, oral prednisolone | - |
| 11 | MUD | 10/10 | - | Lungs | Oral prednisolone, tacrolimus | Oral prednisolone and tacrolimus |
| 12 | MUD | 10/10 | aGVHD grade I  Skin | - genital | Triamcinolone acetonide  Cream, oral cyclosporine | - |
| 13 | MUD | 10/10 | aGVHD grade I  Skin | Skin | Oral prednisolone | - |
| 14 | MUD | 10/10 | aGVHD grade II  Skin, intestinal | Skin | Oral prednisolone, cyclosporine,  Sirolimus, clobetasol mouthwash, | - |
| 15 | SIB | 10/10 | - | Skin,  ~~lever,~~ liver | Oral prednisolone, tacrolimus,  Clobetasol mouthwash, cream | Oral prednisolone and tacrolimus |
| 16 | MUD | 10/10 | aGVHD grade II  Skin, intestinal |  | Oral prednisolone, cyclosporine,  triamcinolone acetonide cream | - |
| 17 | MUD | 9/10 | - | Skin, ~~lever,~~ liver | Tacrolimus, sirolimus, prednisolone | Oral prednisolone and sirolimus |
